# Supplementary material for: Chemical Synthesis, Safety and Efficacy of Antihypertensive Candidate Drug 221s (2,9)
Source: Molecules. 2023 Jun 25;28(13):4975. doi: 10.3390/molecules28134975 (PMC10343876; doi:10.3390/molecules28134975)
Supplement: Supplementary file 1 [file molecules-28-04975-s001.zip › molecules-2436998-supplementary.pdf]

Supplementary materials

Chemical Synthesis, Safety and Efficacy of Antihypertensive Candidate Drug 221s (2,9)

compound 1

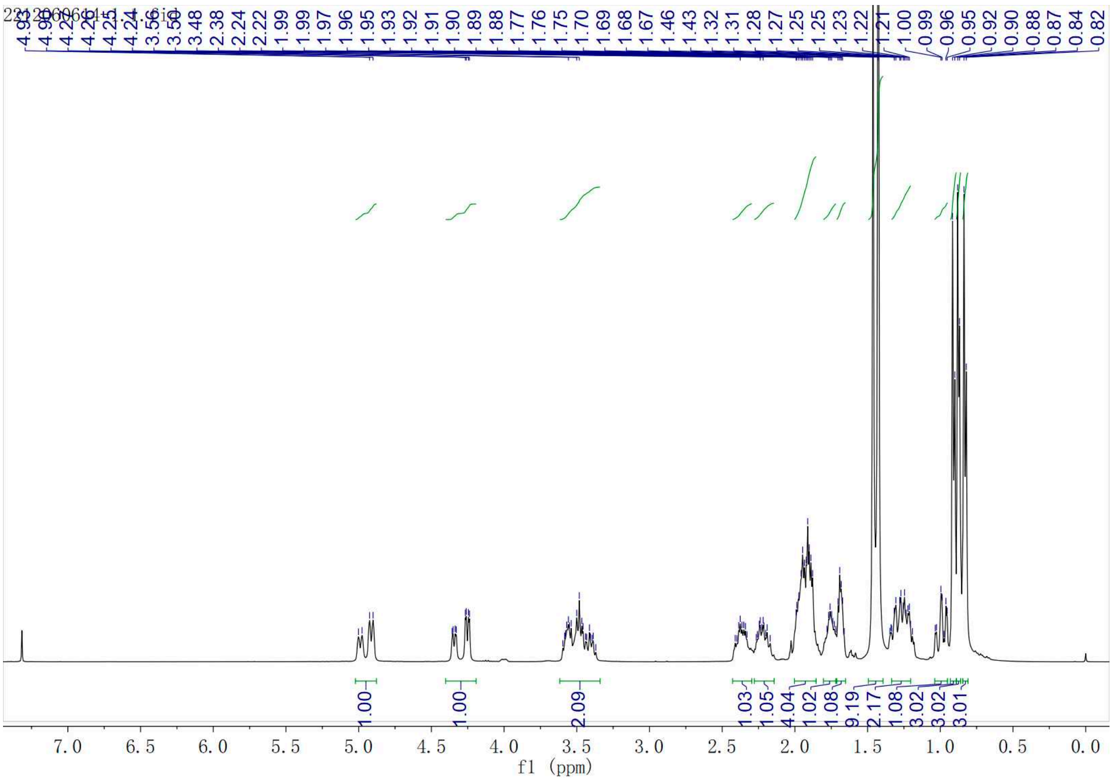

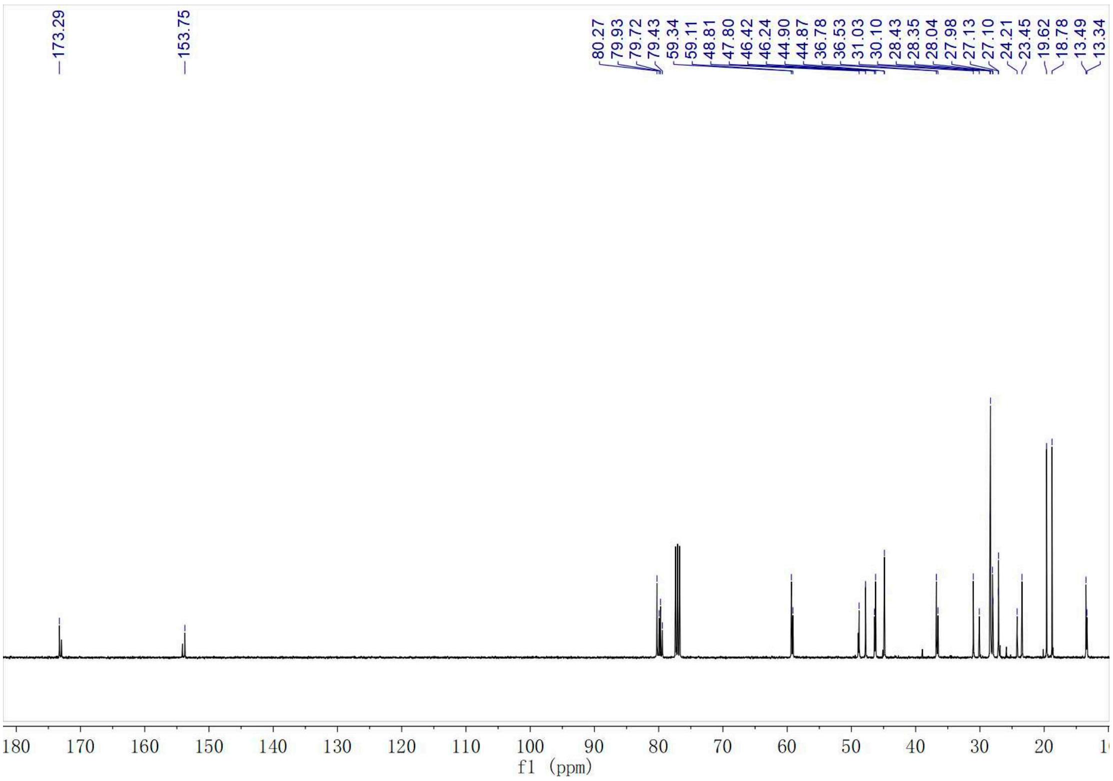

173.29
153.75
80.27
79.93
79.72
79.43
59.34
59.11
48.81
47.80
46.42
46.24
44.90
44.87
36.78
36.53
31.03
30.10
28.43
28.35
28.04
27.98
27.13
27.10
24.21
23.45
19.62
18.78
13.49
13.34
f1 (ppm)

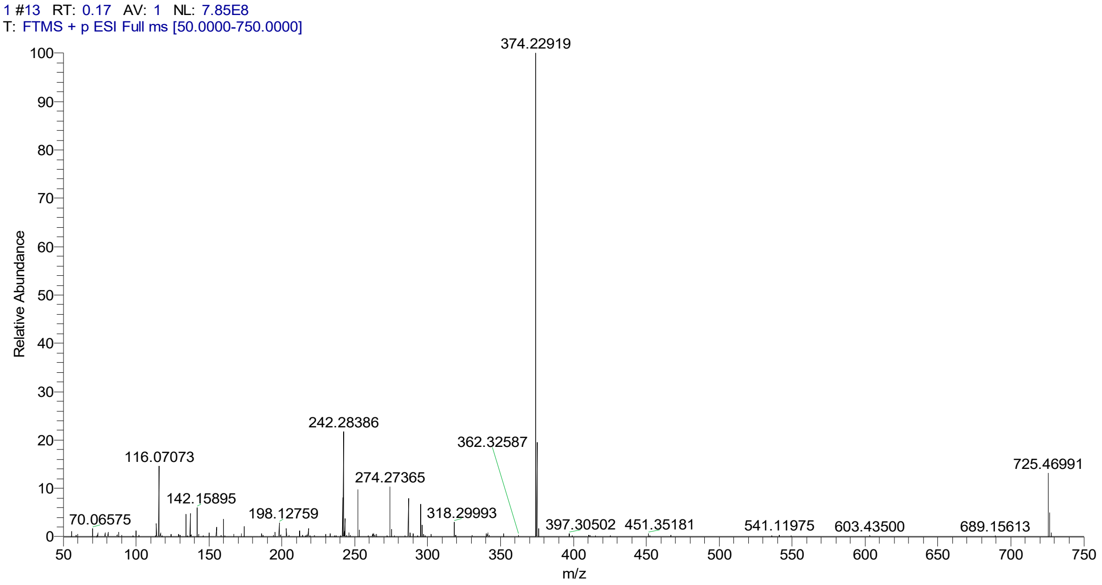

1 #13 RT: 0.17 AV: 1 NL: 7.85E8
T: FTMS + p ESI Full ms [50.0000-750.0000]
Relative Abundance
m/z
374.22919
242.28386
362.32587
116.07073
274.27365
725.46991
142.15895
198.12759
318.29993
70.06575
397.30502
451.35181
541.11975
603.43500
689.15613

compound 3

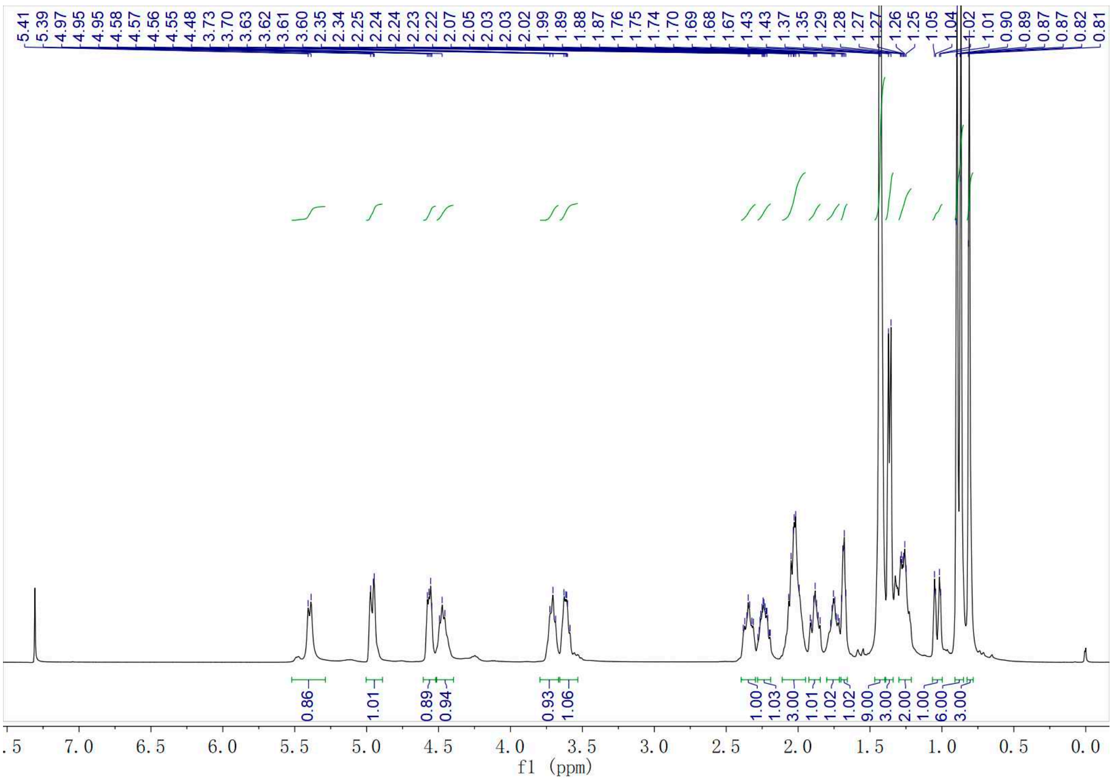
5.41
5.39
4.97
4.95
4.95
4.58
4.57
4.56
4.55
4.48
3.73
3.70
3.63
3.62
3.61
3.60
2.35
2.34
2.25
2.24
2.24
2.23
2.22
2.07
2.05
2.03
2.03
2.02
1.99
1.89
1.88
1.87
1.76
1.75
1.74
1.70
1.69
1.68
1.67
1.43
1.43
1.37
1.35
1.29
1.28
1.27
1.27
1.26
1.25
1.05
1.04
1.02
1.01
0.90
0.89
0.87
0.87
0.82
0.81
0.86
1.01
0.89
0.94
0.93
1.06
1.00
1.03
3.00
1.01
1.02
1.02
9.00
3.00
2.00
1.00
6.00
3.00
.5
7.0
6.5
6.0
5.5
5.0
4.5
4.0
3.5
3.0
2.5
2.0
1.5
1.0
0.5
0.0
f1 (ppm)

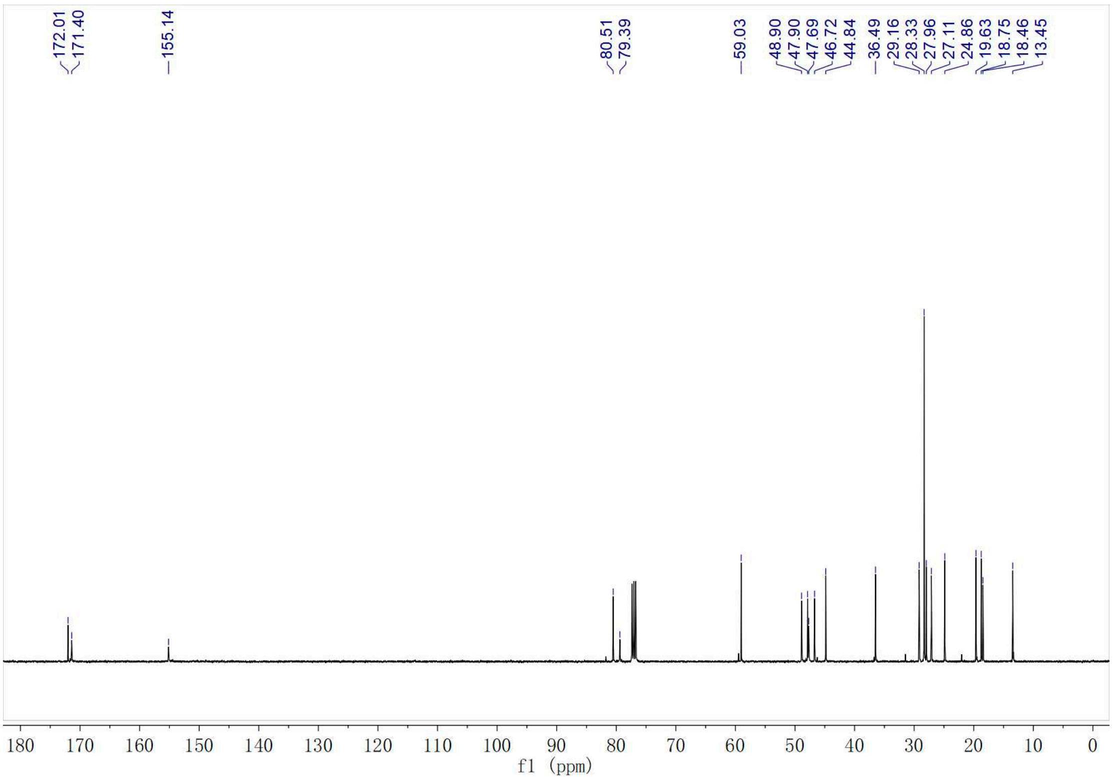
172.01
171.40
155.14
80.51
79.39
59.03
48.90
47.90
47.69
46.72
44.84
36.49
29.16
28.33
27.96
27.11
24.86
19.63
18.75
18.46
13.45
180
170
160
150
140
130
120
110
100
90
80
70
60
50
40
30
20
10
0
f1 (ppm)

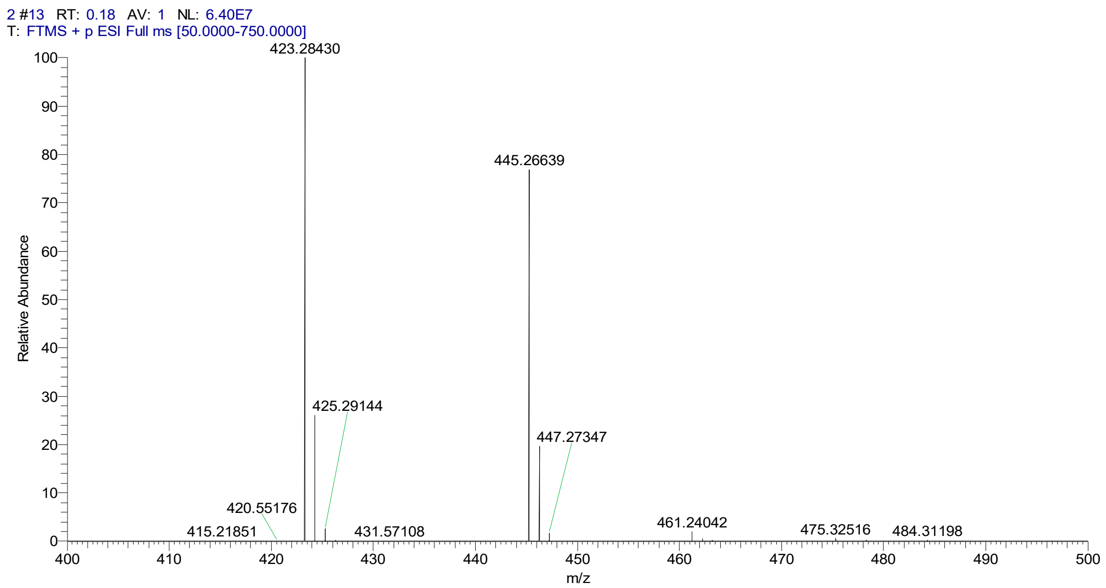
2 #13 RT: 0.18 AV: 1 NL: 6.40E7
T: FTMS + p ESI Full ms [50.0000-750.0000]
423.28430
445.26639
425.29144
447.27347
420.55176
415.21851
431.57108
461.24042
475.32516
484.31198
Relative Abundance
m/z

221s (2,9)

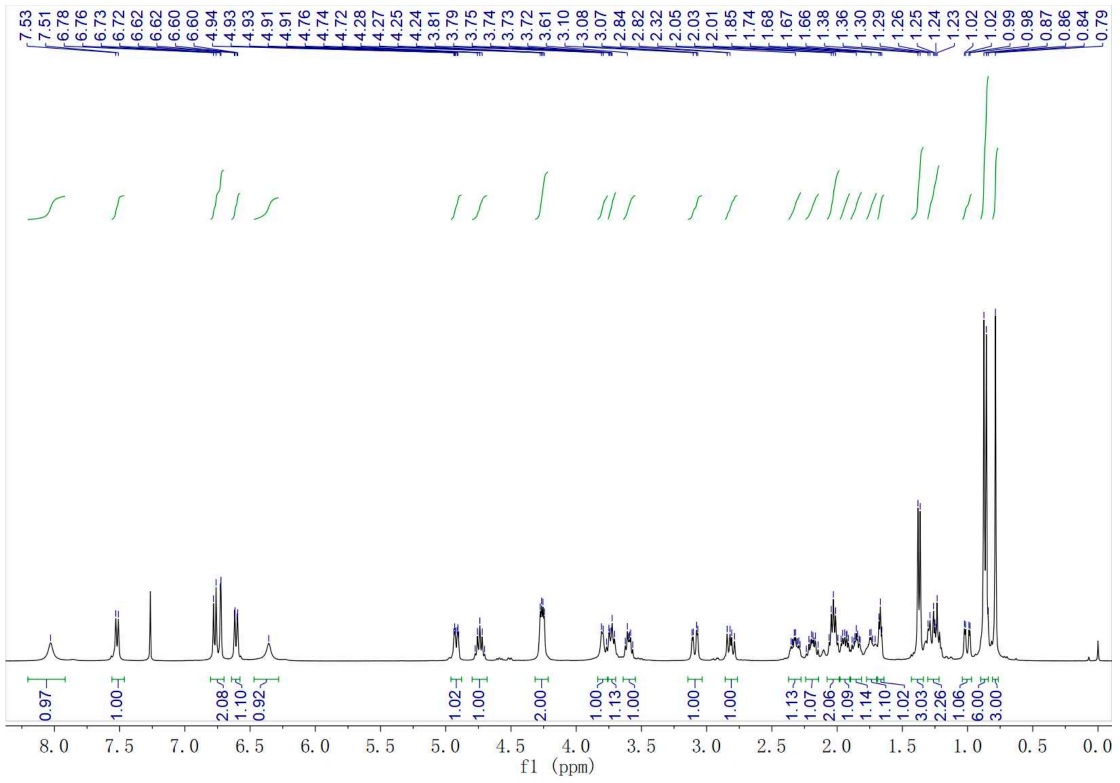
7.53 7.51 6.78 6.76 6.73 6.72 6.62 6.62 6.60 6.60 4.94 4.93 4.93 4.91 4.91 4.76 4.74 4.72 4.28 4.27 4.25 4.24 3.81 3.79 3.75 3.74 3.73 3.72 3.61 3.10 3.08 3.07 2.84 2.82 2.32 2.05 2.03 2.01 1.85 1.74 1.68 1.67 1.66 1.38 1.36 1.30 1.29 1.26 1.25 1.24 1.23 1.02 1.02 0.99 0.98 0.87 0.86 0.84 0.79
0.97 1.00 2.08 1.10 0.92 1.02 1.00 2.00 1.00 1.13 1.00 1.00 1.00 1.13 1.07 2.06 1.09 1.14 1.10 1.02 3.03 2.26 1.06 6.00 3.00
f1 (ppm)

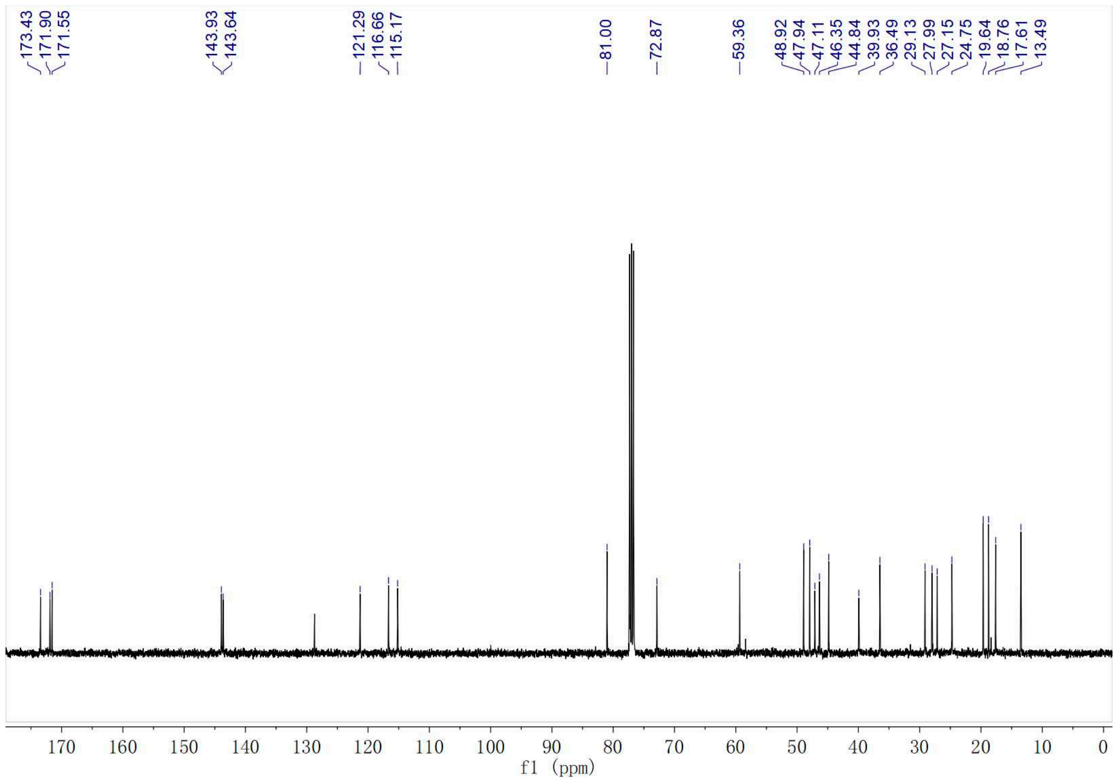
173.43
171.90
171.55
143.93
143.64
121.29
116.66
115.17
81.00
72.87
59.36
48.92
47.94
47.11
46.35
44.84
39.93
36.49
29.13
27.99
27.15
24.75
19.64
18.76
17.61
13.49
170 160 150 140 130 120 110 100 90 80 70 60 50 40 30 20 10 0
f1 (ppm)

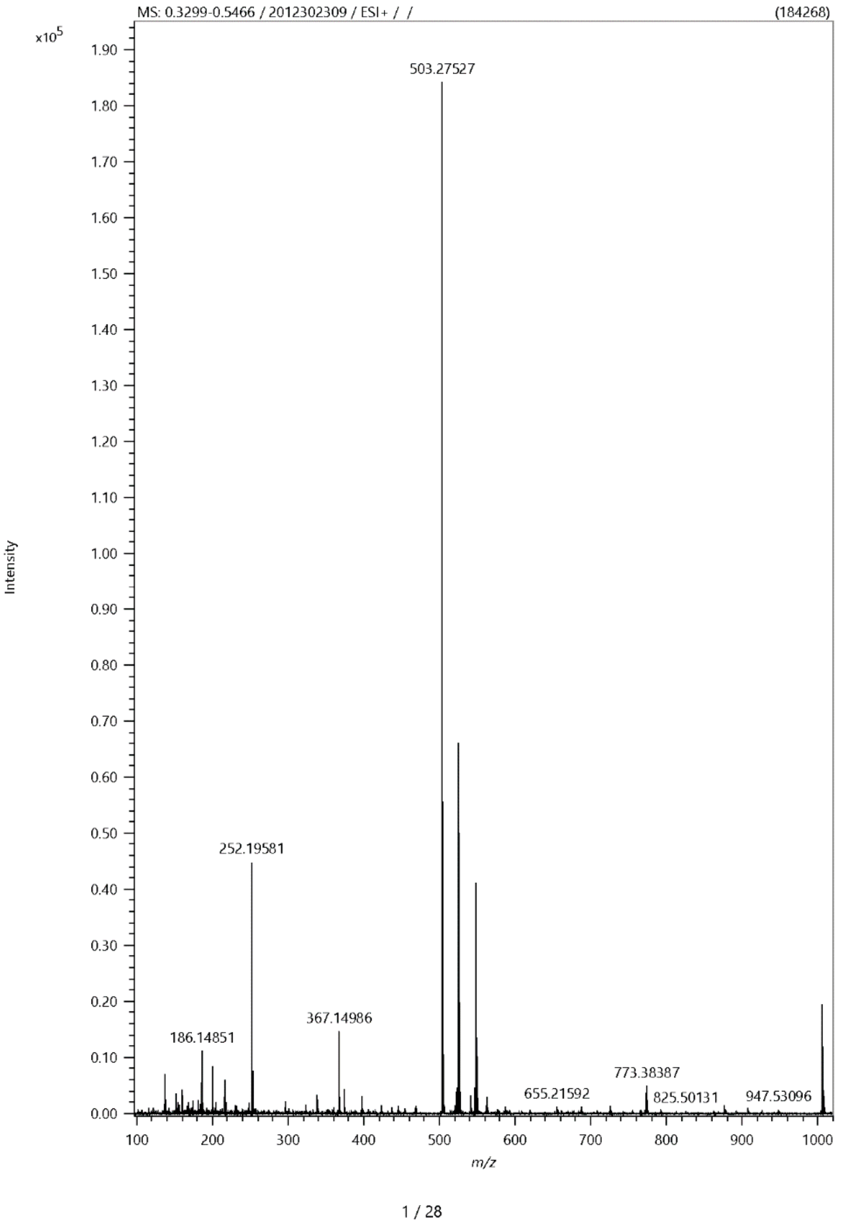

Figure S1. ^{1}HNMR, ^{13}CNMR and mass spectra of compounds

Table S1. Optimal elution gradient of 221s (2,9)

T(min)	A（Acetonitrile）	B（0.2% Formic acid）
0	25	75
9	32	68
10	45	55
40	45	55

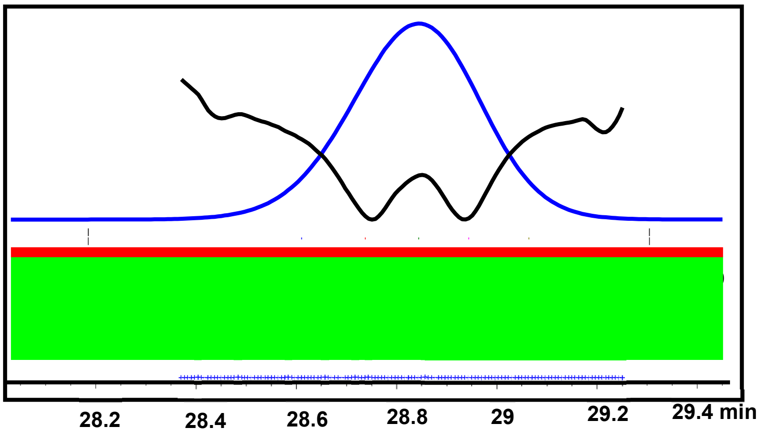

Figure S2. chromatographic peak purity analysis

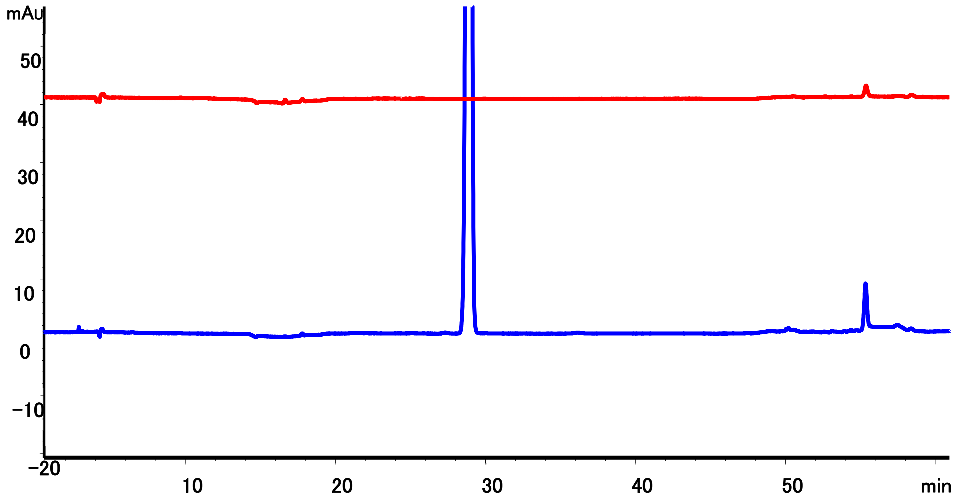

Figure S3. 221s (2,9) chromatogram, sample size: 20uL

Table S2. Normalized method was used to calculate the content of control substance

t(min)	Peak area	Relative content(%)
27.329	5.7	0.074
28.911	7743.6	99.87
36.181	4.2	0.054

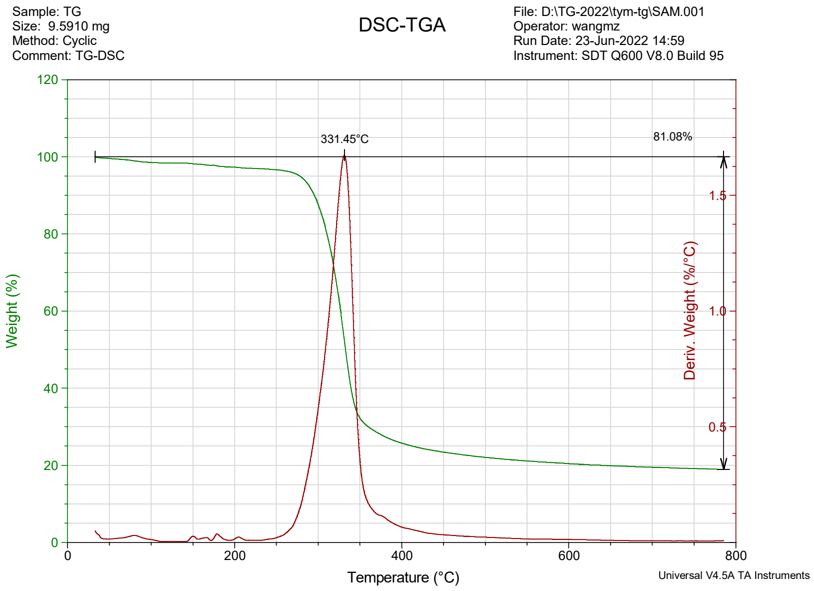

Figure S4. TGA-DTGA curve of 221s (2,9)

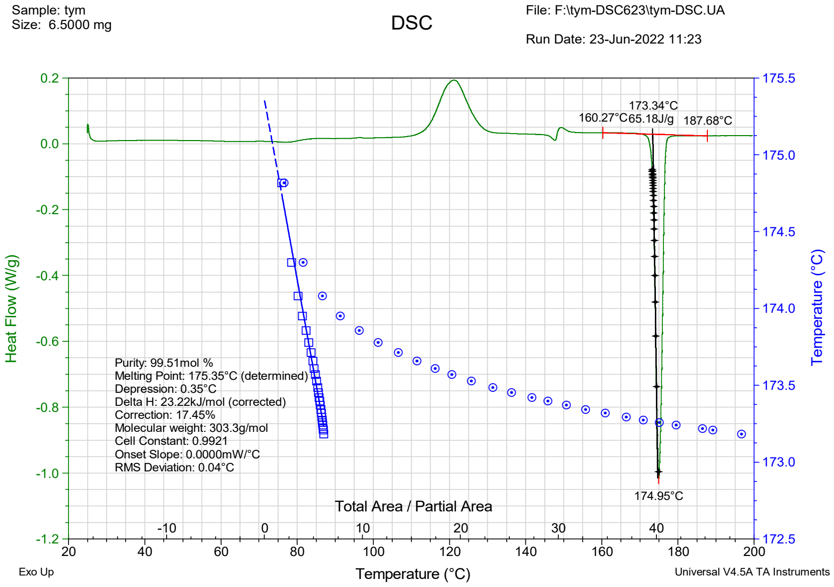

Figure S5. Purity Analysis Results of 221s (2,9)
